# The growth of Ge and direct bandgap Ge_1−*x*_Sn_*x*_ on GaAs (001) by molecular beam epitaxy†

**DOI:** 10.1039/d3ra06774b

**Published:** 2024-01-03

**Authors:** Calbi Gunder, Fernando Maia de Oliveira, Emmanuel Wangila, Hryhorii Stanchu, Mohammad Zamani-Alavijeh, Solomon Ojo, Sudip Acharya, Abdulla Said, Chen Li, Yuriy I. Mazur, Shui-Qing Yu, Gregory J. Salamo

**Affiliations:** a Materials Science and Engineering, University of Arkansas Fayetteville AR 72701 USA calbigunder@gmail.com; b Institute of Nanoscience & Engineering, University of Arkansas Fayetteville AR 72701 USA; c Department of Physics, University of Arkansas Fayetteville AR 72701 USA; d Department of Electrical Engineering, University of Arkansas Fayetteville AR 72701 USA

## Abstract

Germanium tin (GeSn) is a tuneable narrow bandgap material, which has shown remarkable promise for the industry of near- and mid-infrared technologies for high efficiency photodetectors and laser devices. Its synthesis is challenged by the lattice mismatch between the GeSn alloy and the substrate on which it is grown, sensitively affecting its crystalline and optical qualities. In this article, we investigate the growth of Ge and GeSn on GaAs (001) substrates using two different buffer layers consisting of Ge/GaAs and Ge/AlAs *via* molecular beam epitaxy. The quality of the Ge layers was compared using X-ray diffraction, atomic force microscopy, reflection high-energy electron diffraction, and photoluminescence. The characterization techniques demonstrate high-quality Ge layers, including atomic steps, when grown on either GaAs or AlAs at a growth temperature between 500–600 °C. The photoluminescence from the Ge layers was similar in relative intensity and linewidth to that of bulk Ge. The Ge growth was followed by the growth of GeSn using a Sn composition gradient and substrate gradient approach to achieve GeSn films with 9 to 10% Sn composition. Characterization of the GeSn films also indicates high-quality gradients based on X-ray diffraction, photoluminescence, and energy-dispersive X-ray spectroscopy measurements. Finally, we were able to demonstrate temperature-dependent PL results showing that for the growth on Ge/GaAs buffer, the direct transition has shifted past the indirect transition to a longer wavelength/lower energy suggesting a direct bandgap GeSn material.

## Abstract 2

Germanium tin (GeSn) is a tuneable narrow bandgap material, which has shown remarkable promise for the industry of near- and mid-infrared technologies for high efficiency photodetectors and laser devices.

## Graphical abstract

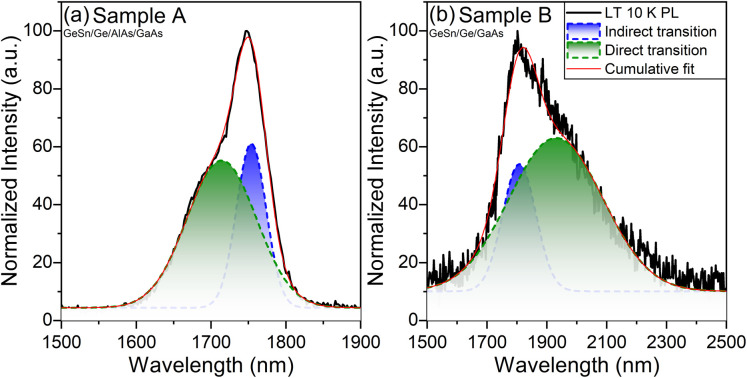

## 1. Introduction

Germanium tin (GeSn) has recently gained increased interest due to the ability to transition from an indirect to a direct bandgap for Sn content of 6 to 11% depending on strain.^1,2^ The tunability of GeSn lends itself to be a great candidate for the next generation of near- and mid-infrared lasers and photodetectors.^1,3–8^ However, achieving the growth of good quality GeSn with significant Sn content is typically challenged by a lattice mismatch between the substrate and GeSn. One possibility is to use a tuneable substrate that allows for a lattice match of different alloy compositions of GeSn. For example, the growth of GeSn of different alloys on InGaAs could provide a lattice matched substrate for different values of Sn content using a corresponding indium content.^9^ As a step in this direction, we first investigated the growth of Ge on GaAs (001) substrates *via* Molecular Beam Epitaxy (MBE) at different growth temperatures, comparing the use of AlAs *versus* GaAs as buffer layers. The temperatures selected for growths were 100 °C, 400 °C, 500 °C and 600 °C, allowing this study to span across a spectrum of growth temperatures that range from low to high in which GeSn and Ge are often grown.^10–16^ The Ge growth was then followed by GeSn on the optimized Ge layers producing GeSn/Ge/GaAs and GeSn/Ge/AlAs/GaAs structures with about 10% Sn composition. The outcome provides an excellent starting point for future work to investigate the growth of high Sn content (>10%) GeSn on tuneable InGaAs and InAlAs relaxed substrates as a function of (In) composition and lattice match.

## 2. Experimental details

Ge films were grown in a Riber-32 MBE system and monitored with *in situ* reflection high-energy electron diffraction (RHEED) operated at 20 keV and 1.6 A with a glancing angle near 1° in reference to the substrate. Undoped GaAs (001) substrates used in these experiments were purchased from Wafer Technology Ltd. Before every growth, the wafers were degassed at 300 °C for 1 hour and then transferred *via* a vacuum transfer line into the main chamber for oxide removal under arsenic flux. The buffer layers of GaAs and AlAs were grown with growth rates of 0.62 ML s^−1^ and 0.6 ML s^−1^ respectively. The arsenic (As) to Ga, Al ratios for growths were maintained near a 15 : 1 and 19 : 1 ratio respectively. All GaAs and AlAs buffer layers that were grown for these experiments had a thickness of 230 nm and 30 nm respectively. For these growths, GaAs was grown at a substrate growth temperature of 585 °C while AlAs was grown at 610 °C. Growth temperatures were monitored *via* a Bandit system which allowed for more precise control of substrate temperature than the typical thermocouple that is located behind the substrate.

For this study, the techniques of X-ray diffraction (XRD), atomic force microscopy (AFM), photoluminescence (PL), scanning electron microscopy (SEM), energy dispersive X-ray spectroscopy (EDS), micro-Raman, and X-ray photoelectron spectroscopy (XPS) were utilized to characterize the samples. XRD was used to investigate crystal quality and strain and was accomplished using a Panalytical X'pert MRD diffractometer utilizing a CuKα1 source of (*λ* = 0.15406 nm). This system makes use of a four-bounce Ge (220) monochromator, a multilayer focusing mirror and a Pixel detector. The tapping mode of AFM (D3100 Nanoscope V made by Bruker) was used to observe the surface morphology. High-quality tips (HQ:NSC15/Al BS made by MikroMasch) were used for AFM measurements. The optical quality of direct and indirect bandgap transitions of Ge and GeSn was investigated using PL spectra obtained through the use of a Bruker IFS 66/S spectrometer together with a 1064 nm laser. The power setting of the 1064 nm laser was maintained at 600 mW for all Ge sample measurements. For the temperature dependent PL study ranging from 10 K to 300 K the 1064 nm laser was also used for both GeSn samples (A) and (B). Sample (A) used 600 mW for the whole range of temperatures while sample (B) used 600 mW for measurements 10 K to 150 K changing to 700 mW for temperatures 200 K, 250 K and finally 800 mW for the measurement at 300 K. The change in power for the final three temperatures for sample (B) was done to maintain the observation of its peak position. The SEM/EDS system used to investigate the surface of GeSn is the FEI Nova Nanolab 200 equipped with a Bruker Xflash 5010 EDX detector. Micro-Raman characterization confirmed strain and composition measurements using a 632.8 nm He–Ne laser and a microscope system (Olympus BX41, lens 100×) in backscattering geometry, with a spectrometer (Horiba Jobin-Yvon LabRam HR) equipped with a thermoelectrically cooled Si charge-coupled device (CCD) detector. XRD simulation was handled through the dynamical theory of X-ray diffraction. For the simulation procedure, the compositionally graded GeSn layer was subdivided into 200 Ge_1−*x*_Sn_*x*_ lamellae. Pseudomorphic growth was assumed based on the measured reciprocal space mapping (RSM) of the GeSn samples. The evolution of Sn composition with depth, and thus the Sn content of each lamella, was observed to be exponential, which is expected for a linear change in the temperature of the Sn effusion cell. XPS was used for two primary functions. First, we made use of its ability to etch the surface of samples, and second, to characterize the composition of the material at periodic steps in the etching process to get the composition at different time etching intervals of the GeSn samples as shown in Fig. S1.† The XPS system used was a PHI Versaprobe 5000 (Physical Electronics Inc., Chanhassen, MN). The XPS main chamber was operated at a base pressure of about 10^−7^ Pa to allow a long mean free path for ejected electrons to reach the detector. XPS sputter-etching made use of an argon ion gun operated with a voltage of 2 kV and a current of 400 nA.

## 3. Results and discussion

### 3.1 Growth of Ge on GaAs (001) substrates using GaAs and AlAs buffer layers

**Fig. 1:**
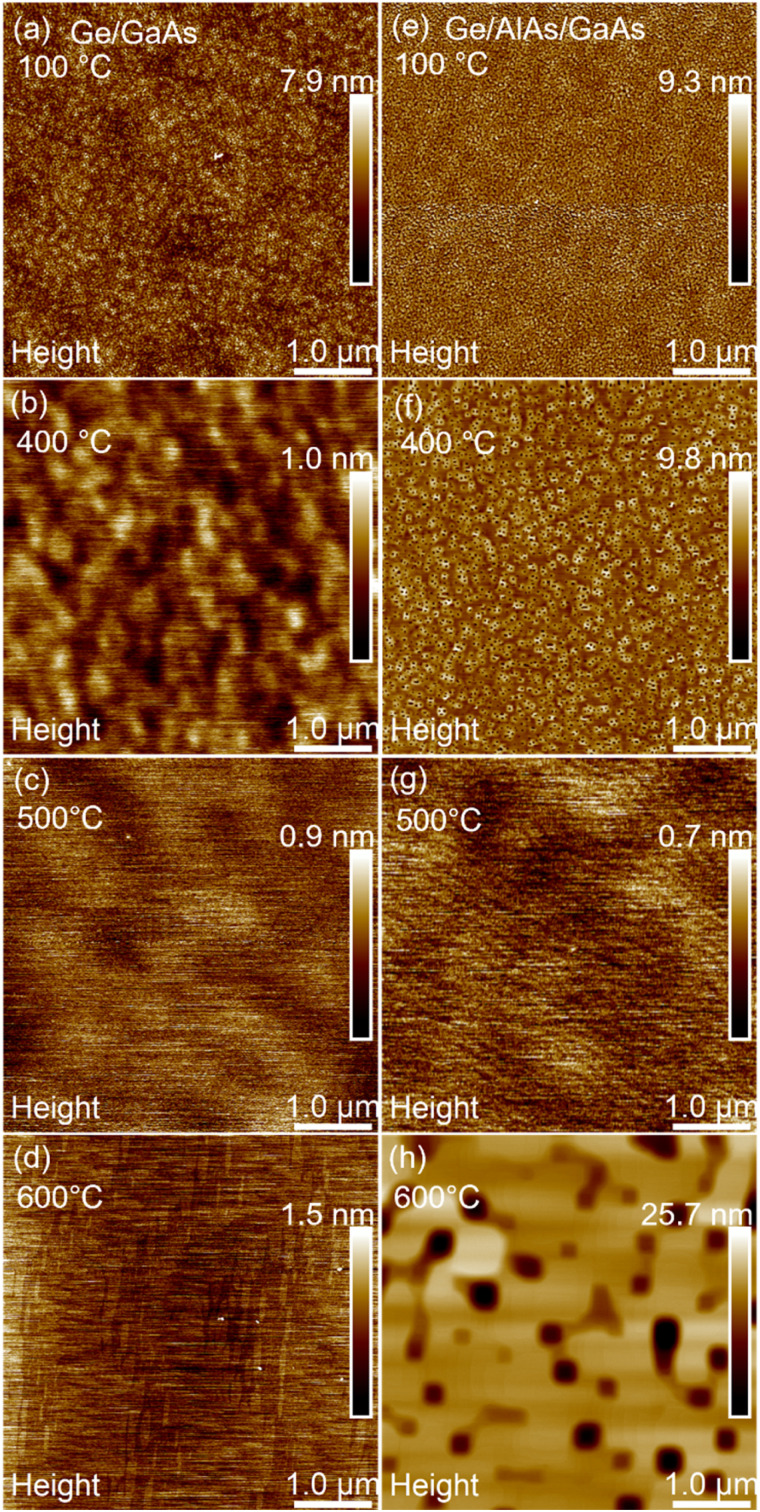
5 × 5 μm^2^ AFM images of Ge surface morphology of samples grown at 100 °C, 400 °C, 500 °C and 600 °C. Images (a–d) consist of the Ge/GaAs series while (e–h) are Ge/AlAs/GaAs series.

We first compared a series of growths at different temperatures, Ge on GaAs *versus* Ge on AlAs/GaAs to find the best surface to grow GeSn. An AlAs interface layer was used to increase wetting on the surface. AFM was primarily used for surface analysis and surface roughness measurements which are displayed in Fig. 1.

**Fig. 2:**
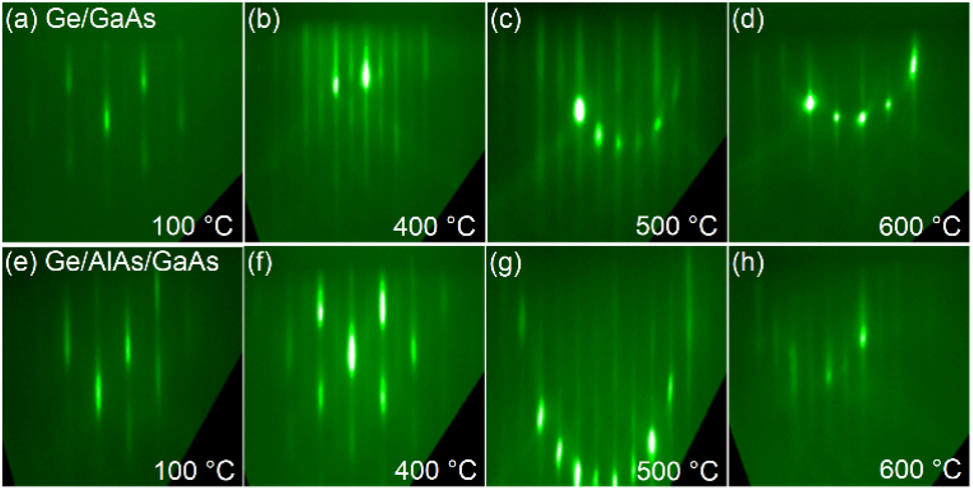
RHEED images taken at the conclusion of growth. Images (a–d) are of Ge on GaAs series while (e–h) are the images taken of the Ge on AlAs series.

Fig. 1(a–d) shows the surface morphology for the growth of Ge on the GaAs series. This set of growths revealed a larger surface roughness at low temperature 100 °C and smoother surfaces at 400 °C and 500 °C with an increase in roughness at 600 °C. The average surface roughness was determined as 0.81 nm, 0.12 nm, 0.11 nm, and 0.17 nm at 100 °C, 400 °C, 500 °C and 600 °C respectively. For the Ge epilayers grown on the AlAs series shown in Fig. 1(e–h), the trend indicates that at 100 °C and 400 °C the surfaces are rough but become extremely smooth at 500 °C. At 600 °C the average surface roughness increases due to the pit formation that is observable across the surface. The average surface roughness of each of the samples was measured to be 0.99 nm, 0.82 nm, 0.08 nm, and 2.06 nm for the growths at 100 °C, 400 °C, 500 °C and 600 °C respectively. Both sample sets, the GaAs buffer series and the AlAs buffer series, achieved the lowest surface roughness at about 500 °C. Layer-by-layer growth was observed for both series of samples. Further information on the surface morphology was observed using reflection high energy electron diffraction (RHEED) images shown in Fig. 2.

For both the Ge on GaAs and Ge on AlAs/GaAs series, RHEED transitions formed a spotty pattern at low temperature (100 °C) to a streaky pattern at higher growth temperatures. The RHEED pattern is consistent with AFM images indicating a rough surface for low-temperature growths and more atomically smooth surfaces at higher temperatures with a sweet spot appearing near 500 °C as shown in Fig. 2(c and g).

**Fig. 3:**
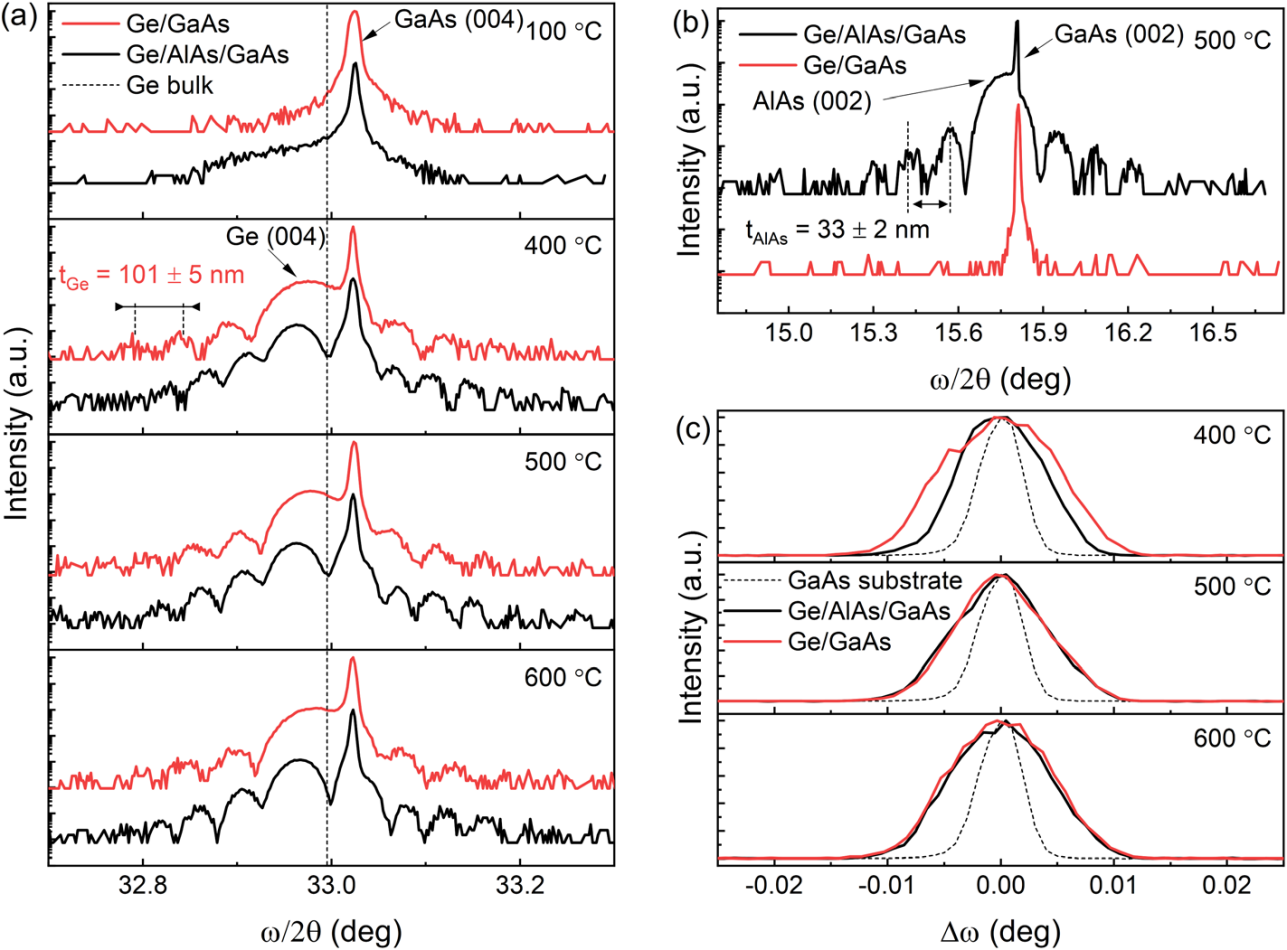
(a) Triple axis X-ray diffraction *ω*/2*θ* scans across (004) GaAs reflection of the samples grown at 100 °C, 400 °C, 500 °C, and 600 °C; (b) *ω*/2*θ* scan across (002) GaAs reflection of samples grown at 500 °C; (c) *ω* scans across Ge peak (004) reflection of samples grown at 400 °C, 500 °C, and 600 °C.

To characterize the crystal quality, the Ge epilayers were also investigated using XRD *ω*/2*θ* scans for the (004) crystal planes of GaAs, AlAs, and Ge shown in Fig. 3(a). For all samples, the Ge peak is seen along with the peak for the GaAs substrate. Due to the slightly larger lattice constant of Ge (*a*_0_ = 0.5658 nm) relative to that of GaAs (*a*_0_ = 0.5653 nm) the Ge peak appears at the lower scattering angle at each growth temperature.^17,18^ The sample grown at 100 °C the Ge peak is observed as a shoulder rather than a distinct peak suggesting the temperature for the growth is too low and is forming an amorphous low-quality material. In addition, the Ge peak is also left-shifted relative to the position of bulk Ge (vertical dashed line), which indicates a larger lattice constant in the out-of-plane direction due to compressive in-plane strain. Well-defined Pendellösung fringes can also be observed on the spectra from the Ge epilayers grown above 100 °C. Growth at 100 °C is too low of a temperature to allow mobility of atoms at surface to form a nice smooth thin film capable of generating interference fringes. The distance between the fringes confirms an average sample Ge layer thickness of *t*_Ge_ = 103 ± 5 nm, according to eqn (1),^19^1
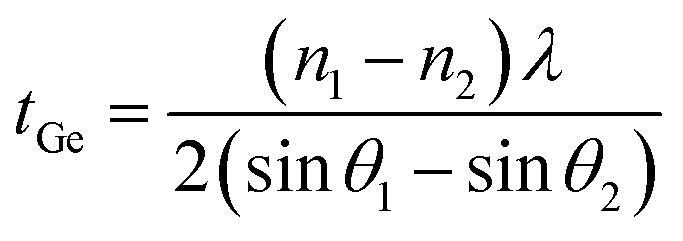
where *n* is the peak order, *θ* is the peak position, and *λ* is the X-ray wavelength. The thickness of one AlAs buffer layer (33 ± 2 nm) was determined similarly using eqn (1) by performing the *ω*/2*θ* scanning for the (002) planes which agrees well with the original growth target of 30 nm. The error was determined from the standard deviation between the measurements of multiple thickness fringe measurements. The (002) is a forbidden reflection due to interference for Ge, and thus, the Ge peak is absent in the diffraction pattern. This is evident in Fig. 3(b), where only the peaks from the GaAs substrate and AlAs buffer layer can be seen.

The structural quality of the Ge layers for different growths was compared by analyzing the full-width-at-half-maximum (FWHM) of *ω* scans for the (004) Ge reflection shown in Fig. 3(c). The XRD curves for the samples grown at 100 °C are not used for comparison since the Ge peak is not clearly defined on the *ω*/2*θ* scans in Fig. 3(a). The FWHMs for the samples grown at 400 °C, 500 °C, and 600 °C are listed in Table 1.

**Table 1:** Ge layer thickness and FWHM from Ge (004) *ω* scans

Temperature (°C)	Ge/AlAs/GaAs		Ge/GaAs	
	Thickness (±5 nm)	FWHM (arcsec)	Thickness (±5 nm)	FWHM (arcsec)
400	107	31.3	101	43.0
500	103	34.9	110	34.3
600	97	37.1	101	38.4

**Fig. 4:**
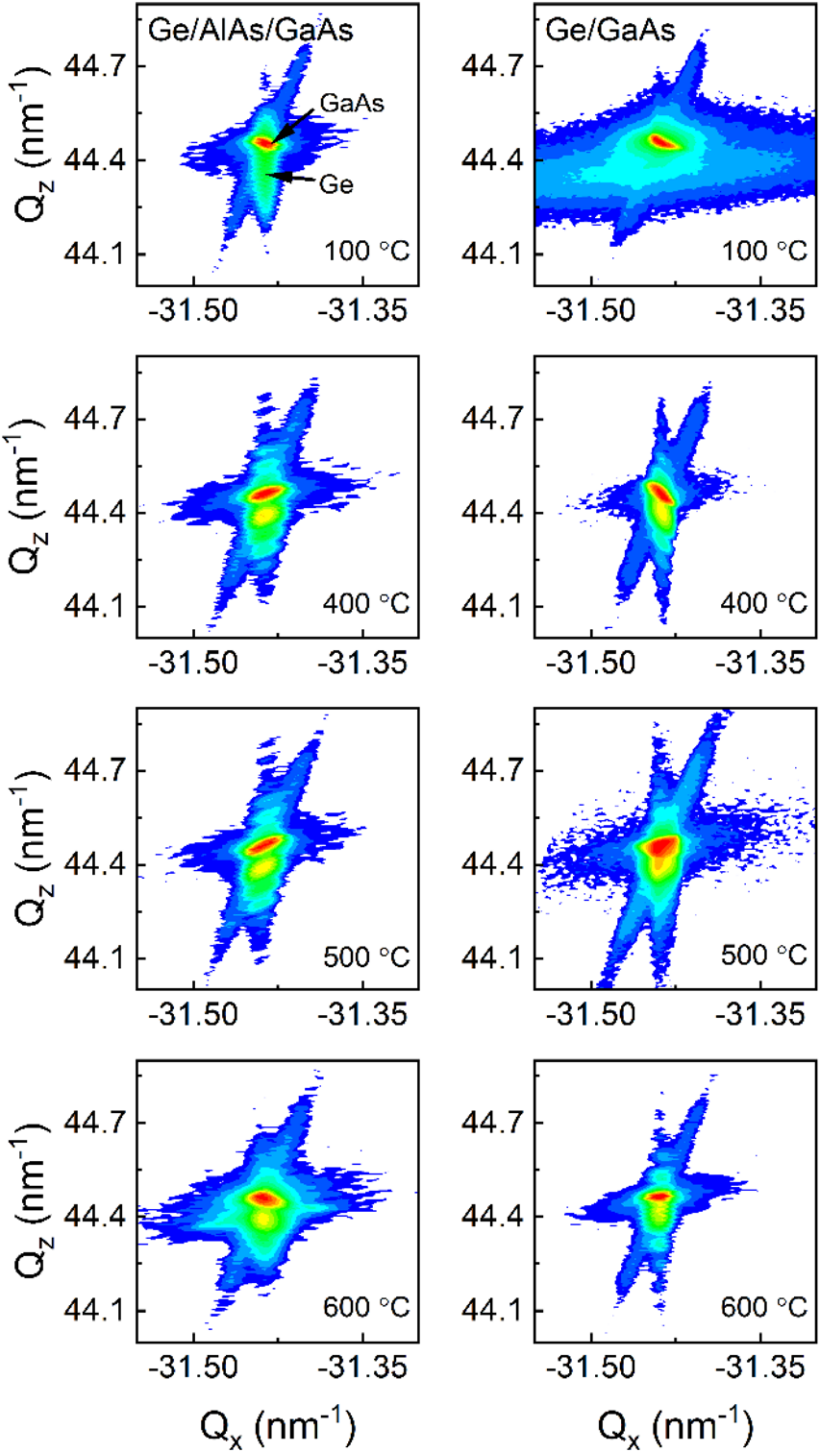
XRD RSM measured from the 2̄2̄4 crystal planes.

All samples exhibit a narrow linewidth with the AlAs series indicating a higher quality. The information about the in-plane strain state was additionally extracted from the XRD RSM of the 2̄2̄4 reflection as shown in Fig. 4. In particular, the *Q*_*x*_ position of the Ge peaks is very similar to that of the GaAs substrate, which indicates a fully strained growth of Ge (and Ge/AlAs) on GaAs has occurred. For tetragonally distorted cubic crystals, the relationship between the *Q*_*x*_, and in-plane (*a*_‖_) lattice parameter and a (*hkl*) reflection is,2
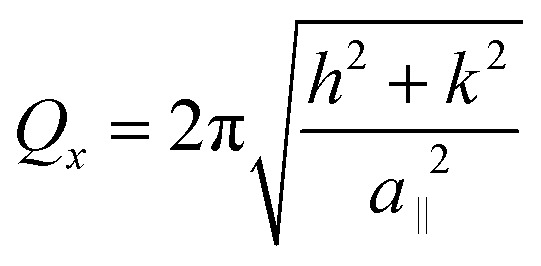
and the strain *ε*_*xx*_ is given by,3
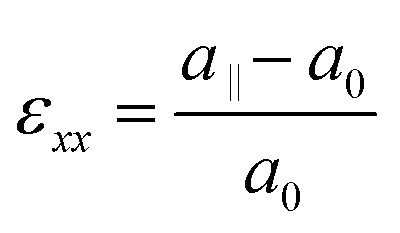
where *a*_0_ represents the lattice parameter of Ge (*a*_0_ = 0.5658 nm) and *a*_‖_ comes from the calculated in-plane lattice parameter from eqn (2). According to the Ge peak position on the RSM and eqn (2) and (3), the Ge layer is compressively strained for all samples with an average of *ε*_*xx*_ = −9.1 ± 0.3 × 10^−4^ for Ge/AlAs/GaAs and *ε*_*xx*_ = −9.3 ± 0.3 × 10^−4^ for Ge/GaAs.

**Fig. 5:**
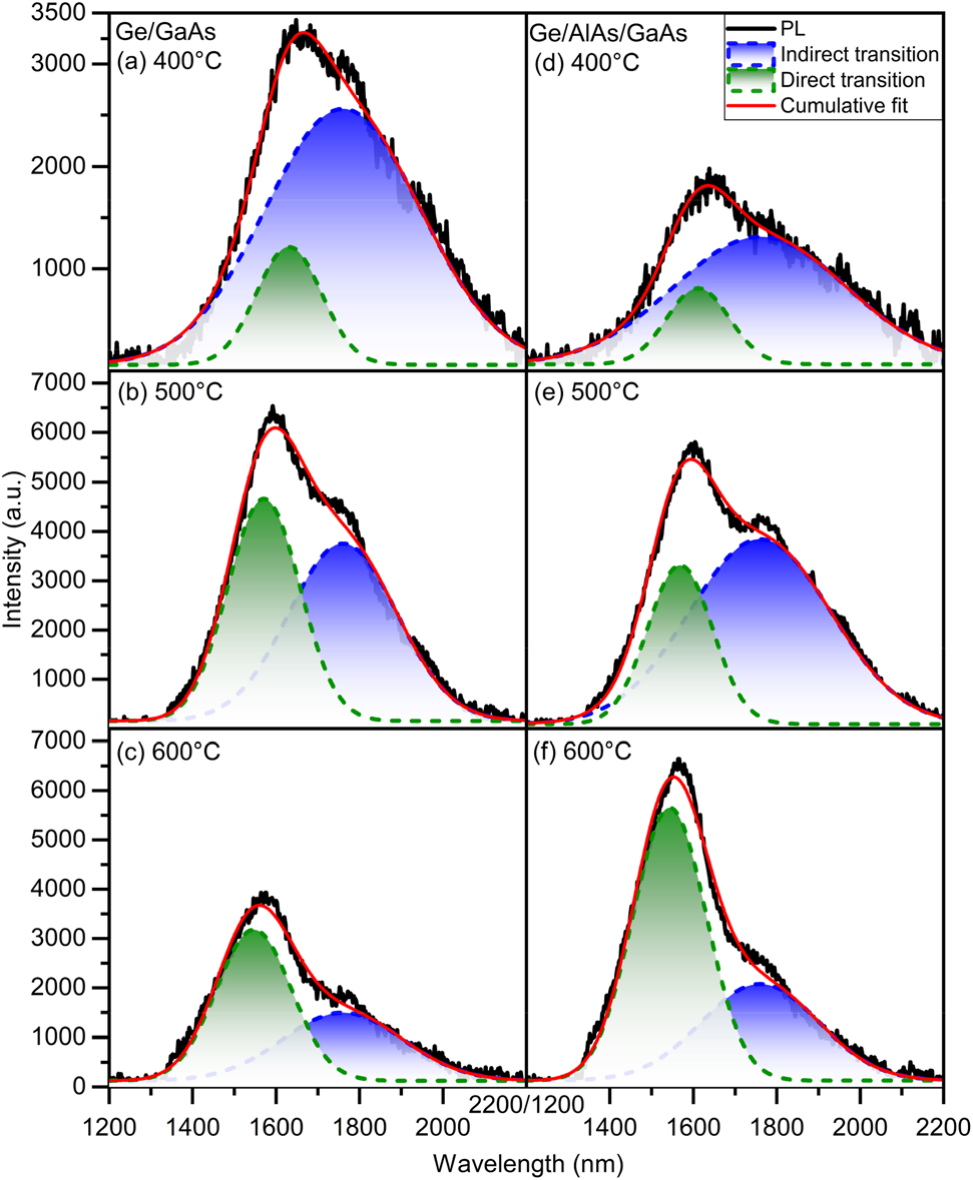
Room temperature PL of Ge on GaAs (a–c) and AlAs (d–f). All plots make use of multi-peak Gaussian fits to the PL spectra data to aid in identifying the contributions from the direct and indirect transitions.

The optical quality of the Ge films can also be estimated using the PL technique. To compare the optical quality of Ge on GaAs to Ge on AlAs/GaAs, the PL was acquired using a 1064 nm laser at a power of 600 mW. In Fig. 5 the plots (a–c) are for the Ge/GaAs series while (d–f) are for the Ge/AlAs series. With the exception of the samples grown at 100 °C, which exhibited weak PL, the PL spectra of the samples grown at higher temperatures were deconvolved by fitting to Gaussian functions. The multi-peak Gaussian fitting was used to examine the Ge direct and indirect transition contributions. The PL emission associated with the indirect transition was fixed at 1760 nm, due to the very low measured strain in the Ge films, which is consistent with the reported literature.^20,21^Table 2 shows the energy associated with the direct peak for each sample in both series.

**Table 2:** PL multi-peak Gaussian fitting with locked indirect peak position at 1760 nm

Temperature (°C)	Ge/AlAs/GaAs	Ge/GaAs
	Direct peak position (nm)	Direct peak position (nm)
400	1611.33 ± 1.96	1630.78 ± 1.88
500	1567.98 ± 0.68	1570.61 ± 0.61
600	1543.06 ± 0.47	1546.56 ± 0.68

In Fig. 5, the PL results are shown to be consistent with both AFM and XRD, favoring a growth temperature at about 500 °C for both series. To better compare the PL emission efficiency among the set of samples it is necessary to account for the effective photoexcitation of the Ge films along the thicknesses, *t*_S_, presented in Table 1. Given that the penetration depth, *t*_B_, of the laser 1064 nm in Ge is about 700 nm, with an absorption coefficient, *α*, on the order of 10^4^ cm^−1^,^22,23^ the relative photoexcitation of each sample, *φ*_s_, compared to bulk Ge can be represented by:4
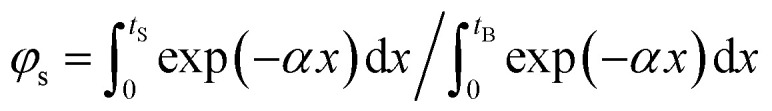
Here the integral is defined along the depth, *x*, into the Ge film. Therefore, by computing the ratio, *η*_s_, between the integrated PL intensity of each Ge film relative to bulk Ge along the entire spectral range displayed in Fig. 5, the effective PL emission efficiency weighted by the relative photoexcitation of each sample can be represented by:

5 *η*^effective^ = 100 × *η*_s_/*φ*_s_

The calculated values of *η*^effective^ for each sample are presented in Table 3. Clearly, for both sets of samples, the growth temperature of 500 °C favors the PL emission efficiency, with similar contributions from direct and indirect transitions, leading to a PL emission intensity that is about a third of that seen for bulk Ge (30 ± 1%). This is an indication of high Ge thin film optical quality.

**Table 3:** Effective PL emission efficiency, *η*^effective^ (±0.5%)

Temperature (°C)	Ge/AlAs/GaAs	Ge/GaAs
400	11.0	19.8
500	30.8	29.1
600	28.9	18.1

### 3.2 Growth of GeSn on Ge buffers

**Fig. 6:**
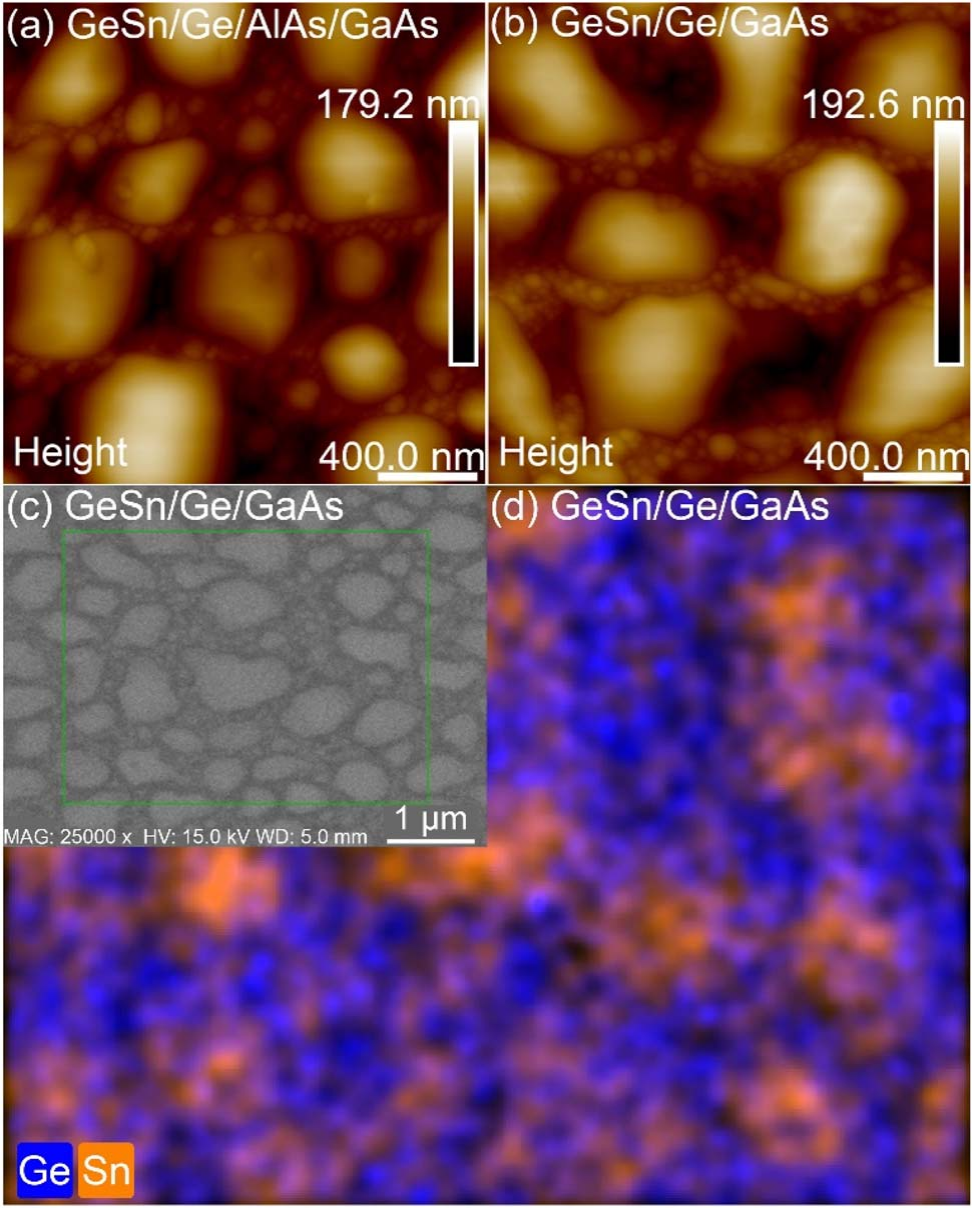
Images (a) and (b) are AFM of GeSn grown on AlAs and GaAs series respectively while image (c) is SEM and (d) is EDS of GeSn on the GaAs series.

Based on the results for Ge on GaAs and AlAs series we investigated the growth of GeSn with Ge buffers grown at 500 °C on GaAs and AlAs as discussed above in Section 3.1. The growth utilized both a gradient in Sn composition and a gradient in substrate temperature set to decrease from 200 °C down to 50 °C. A manipulator ramp rate of 10 °C min^−1^ was set however due to radiative heating from the Ge and Sn cells and the fact the MBE manipulator head does not have active cooling the actual cooling is non-linear. Due to this heating issue the final thermocouple temperature ended near 90 °C ± 5 °C. The gradient in growth temperature was done to minimize the impact on the substrate surface temperature increase during growth as an attempt to stay below the eutectic temperature required to grow its corresponding GeSn composition. The Sn cell gradient was used to increase the Sn content while attempting to manage strain and thus maximize the GeSn composition. For both GeSn growths AFM, XRD, PL, SEM, EDS, micro-Raman and XRD analyses were used to obtain a comparable analysis of both surface morphology and GeSn compositions between the two samples. Fig. 6 shows the AFM (a and b), SEM (c) and EDS (d) images associated with the two GeSn growths. The average surface roughness observed by AFM is measured to be 36.7 nm on the GaAs system while on the AlAs system the surface roughness is 24.2 nm. SEM/EDS images reveal that the mounds on the surface correlate to Sn droplets.

**Fig. 7:**
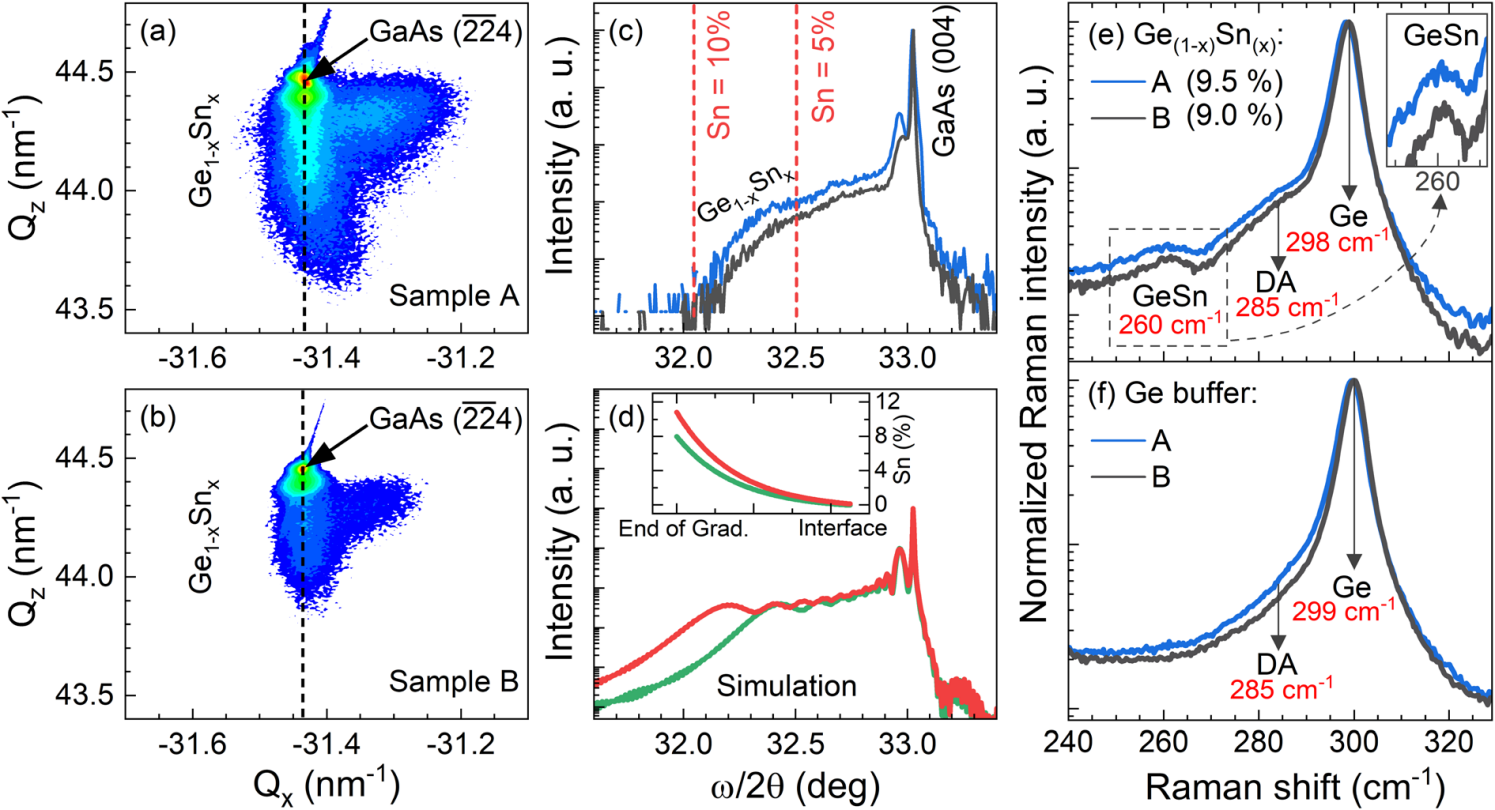
(a) XRD RSM measured from the 2̄2̄4 crystal planes of sample A (GeSn/Ge/AlAs/GaAs) and (b) B (GeSn/Ge/GaAs); (c) *ω*/2*θ* spectrum with two compositions shown assuming fully strained and (d) simulation of each sample, displaying the calculated Sn profile as an inset. (e) Raman spectrum of each sample and (f) of their respective buffer.

The compositions of the two GeSn samples were also investigated through the use of measured and simulated XRD data. Using XRD, we evaluated *ω*/2*θ* scans and RSM measurements to determine both strain and composition. Fig. 7 shows the XRD and micro-Raman measurements for sample A (GeSn on Ge/AlAs) and sample B (GeSn on Ge/GaAs). The compositionally graded Ge_1−*x*_Sn_*x*_ epilayer can be seen on the 2̄2̄4 RSMs in Fig. 7(a and b) as a diffraction streak extending downward from the GaAs peak. The vertical alignment of the substrate and epilayer diffraction indicates the pseudomorphic growth of the Ge_1−*x*_Sn_*x*_ epilayer. Additionally, a low-intensity diffraction area is also seen on the right side of the elongated streak, indicating a GeSn relaxed region with lower Sn composition, at the top of the sample. The *ω*/2*θ* spectrum shown in Fig. 7(c), the X-ray scattering from the compositionally graded Ge_1−*x*_Sn_*x*_ epilayer results in a similar broad composition extending toward lower angles, in accordance with a gradually increasing lattice parameter of the Ge_1−*x*_Sn_*x*_ alloy. The XRD simulations shown in Fig. 7(d), reveal a graded growth of Sn composition from 0 to 11%. The incorporation of Sn into the Ge lattice of the Ge/AlAs and Ge/GaAs systems is also evidenced in the Raman spectra shown in Fig. 7(e) by its characteristic mode of GeSn near 260 cm^−1^, as well as by the intensification of the disorder activated (DA) mode.^24,25^ Using the deformation potentials *a*_ω_ = −84 ± 8 cm^−1^ and *b*_ω_ = −491 ± 52 cm^−1^ of the Raman line shape of the Ge host lattice exhibiting a Raman shift of Δ*ω* from *ω*_0_ = 300.4 ± 0.9 cm^−1^, eqn (6) provides a maximum average Sn content *x* of 9.5 ± 0.2% and 9.0 ± 0.2% for samples A and B, respectively, which is within the composition range obtained from XRD.6
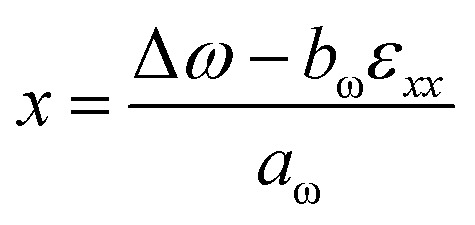


In order to get optical PL spectra of both GeSn samples near the 10% Sn composition mark, XPS sputter-etching was used to remove excess Ge/Sn that was not part of the primary GeSn gradient crystal structure. This excess Ge/Sn took the form of a relaxed low composition GeSn mixed layer with Sn segregated droplets. This is evidenced by the RSM mapping that is presented in Fig. 7(a and b)*via* an elongation pattern extending toward *Q*_*x*_ = −31.2. The Sn-segregated droplets are evidenced in Fig. 6(c and d) through the SEM/EDS characterization of the surface. Although the use of gradients of Sn flux and substrate temperature during growth reduces Sn droplets on the surface. This excess roughness/material is related to the shape and magnitude of the gradient which is not optimized for the material to consume the Sn during growth. The Sn on the surface can potentially be eliminated or at least reduced using a smaller Sn cell temperature range. A further interesting approach to eliminate Sn on the surface is to consider a logarithmic change to the Sn cell ramp rate to control the nonlinear increase in Sn flux. Both approaches are now under study in our lab.

**Fig. 8:**
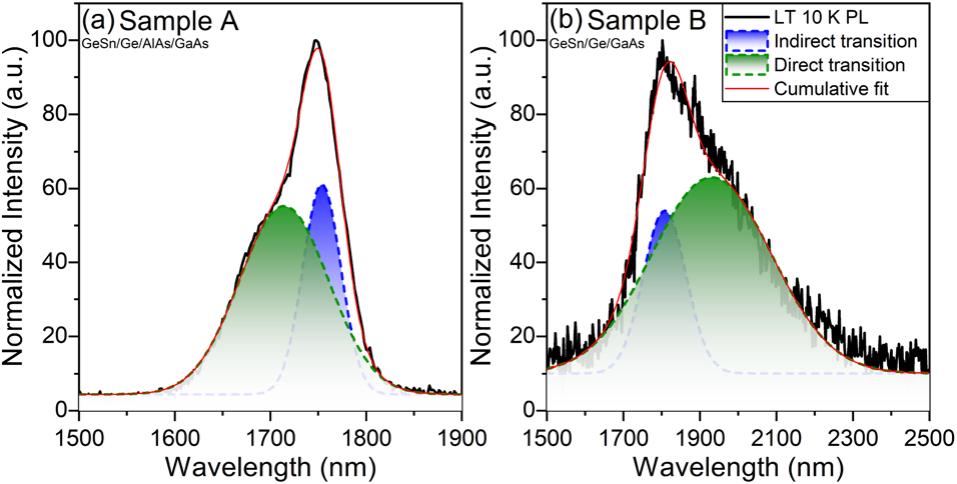
Low temperature normalized 10 K PL of GeSn samples with gaussian fittings. Image (a) is of GeSn/Ge/AlAs/GaAs while (b) is of GeSn/Ge/GaAs.

**Fig. 9:**
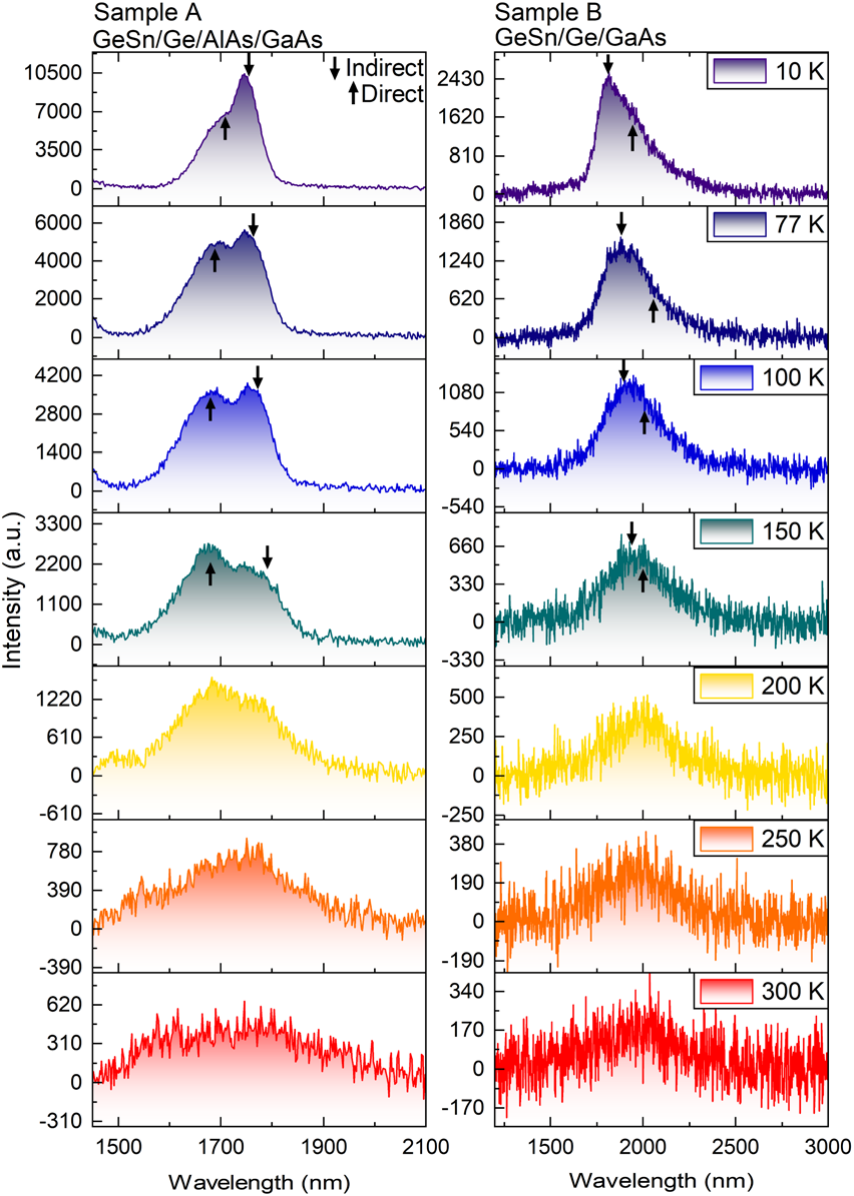
Temperature dependence PL spectra of samples A and B ranging from 10 K to 300 K. Gaussian fittings were used to give an indication of where the direct and indirect bandgap transitions are likely located.

The PL optical information of the GeSn gradient is presented in Fig. 8 and 9. The measurements were taken at 10 K to investigate the presence of the direct bandgap transition, using a 1064 nm laser. In both samples, the direct and indirect bandgap transitions are observable. This type of low temperature (LT) behavior is also documented by Stange *et al.*,^26^ and Ghetmiri *et al.*^27^ The shape of the LT PL emissions and where the shoulder occurs can give insight as to the location of the direct transition. By using Gaussian fittings, it is possible to evidence the individual contributions to the total cumulative PL.^28^ In both cases, the indirect bandgap transition forms the sharp edge of the PL spectrum, while its broadening is attributed to the direct transition at low temperature. In Fig. 8 it is seen that the linewidth of the indirect bandgap transition at 10 K is narrower, while the direct transition broadens the PL emission causing overlap in the spectrum.

A temperature dependence study was done to investigate how the PL bandgap transitions change as a function of temperature as shown in Fig. 9. It is observed in both cases the intensity of the indirect transition decreases until the direct transition becomes more prominent. In sample A the cumulative PL shift is observed toward the shorter wavelength/higher energy while sample B shifts toward the longer wavelength/lower energy. In sample A the PL after 200 K predominately changes into one broader peak suggesting above 200 K sample A becomes more direct due to the large spectral overlap and the appearance of one virtual peak. The key takeaway between both GeSn samples is that sample B (GeSn/Ge/GaAs) demonstrated a direct transition shifted to a longer wavelength/lower energy than the indirect transition. This result suggests that sample B (GeSn/Ge/GaAs) is completely a direct bandgap material while the results from sample A (GeSn/Ge/AlAs/GaAs) suggest it is pseudo-direct at LT 10 K due to the spectrum overlap of the direct over the indirect transition while becoming more direct from 200 K and above in temperature.

## 4. Conclusions

It was demonstrated that the growth of germanium on both GaAs and AlAs series are of high optical quality and are optimized near 500 °C. The FWHM values measured by omega scans of both Ge sample sets indicate that a higher quality Ge layer was produced on AlAs than the direct growth on GaAs. It was also seen that the surfaces of both samples are smooth with a slight improvement on the AlAs system, revealing atomic steps on the surface. The high-quality Ge growth was also supported by the similarity between the photoluminescence of the Ge layers to that of bulk Ge, in both intensity and linewidth. The GeSn gradient growths on both series of Ge/GaAs and Ge/AlAs/GaAs samples exhibited a maximum GeSn composition of about 10%. Another key highlight is the PL spectra of both GeSn samples. The PL spectra taken at 10 K suggests that sample B (GeSn/Ge/GaAs) is a direct bandgap material due to its direct transition occurring at a longer wavelength/lower energy than its indirect transition. In the case of sample A (GeSn/Ge/AlAs/GaAs), the PL spectrum indicates a pseudo-direct bandgap material due to the large spectral overlap of the direct transition over the indirect transition. The temperature dependent study of sample A suggests that the bandgap emission of this GeSn film becomes more direct at temperatures above 200 K. These results are encouraging for future studies of GeSn on lattice matched relaxed InGaAs and InAlAs substrates to achieve high quality GeSn films with potentially higher Sn content. Using a gradient Sn deposition can help with achieving a higher Sn content without the introduction of relaxation and its corresponding dislocations, yet producing a direct bandgap material.

## Author contributions

Calbi Gunder: project administration, methodology, investigation, data curation, formal analysis, visualization, conceptualization and writing – original draft. Fernando Maia de Oliveira: data curation and formal analysis. Emmanuel Wangila: data curation and writing – review & editing. Hryhorii Stanchu: data curation and formal analysis. Mohammad Zamani-Alavijeh: data curation and writing – review & editing. Solomon Ojo: data curation. Sudip Acharya: data curation. Abdulla Said: data curation. Chen Li: resources. Yuriy I. Mazur: formal analysis and writing – review & editing. Shui-Qing Yu: project administration and funding acquisition. Gregory J. Salamo: project administration, conceptualization, funding acquisition and writing – review & editing.

## Conflicts of interest

There are no conflicts to declare.

## Supplementary Material

RA-014-D3RA06774B-s001
