# Supplementary material for: The growth of Ge and direct bandgap Ge1−xSnx on GaAs (001) by molecular beam epitaxy
Source: RSC Adv. 2024 Jan 3;14(2):1250–7. doi: 10.1039/d3ra06774b (PMC10762728; doi:10.1039/d3ra06774b)
Supplement: RA-014-D3RA06774B-s001 [file RA-014-D3RA06774B-s001.pdf]

Electronic Supplementary Material (ESI) for RSC Advances.
This journal is © The Royal Society of Chemistry 2023

Supplementary information

XPS measurements:

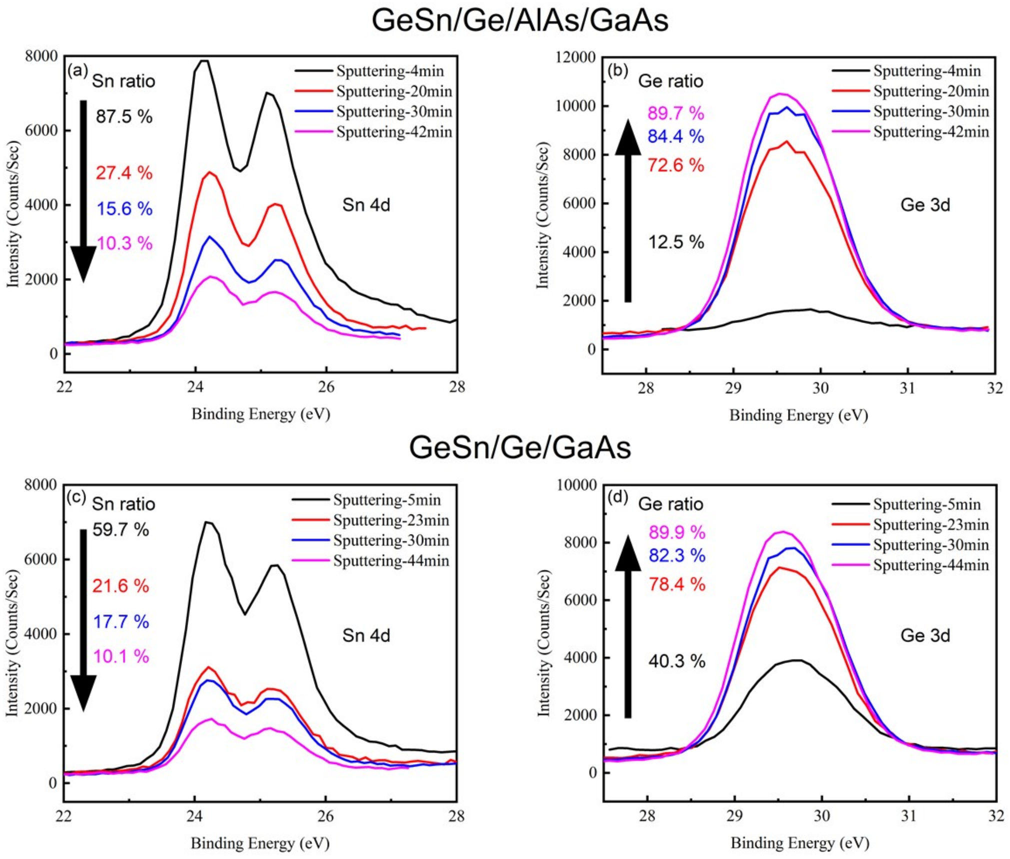

Figure S1: Sn 4d and Ge 3d binding energy peaks of GeSn/Ge/AlAs/GaAs in panels (a) and (b), and GeSn/Ge/GaAs in panels (c) and (d) after different sputtering times. The ratios in the plots display the concentrations of Ge and Sn after different times of Ar ion sputtering, and the arrow shows the change in the concentration throughout etching.

XRD out-of-plane relationships:

The out-of-plane lattice parameter is represented by $a_{\perp}$ while ε_z represents the out-of-plane strain.

$$Q_z = \frac{2\pi l}{a_{\perp}} \qquad (S1)$$

$$\varepsilon_z = \frac{a_{\perp} - a_0}{a_0} \qquad (S2)$$
